# The presence of the ancestral insect telomeric motif in kissing bugs (Triatominae) rules out the hypothesis of its loss in evolutionarily advanced Heteroptera (Cimicomorpha)

**DOI:** 10.3897/CompCytogen.v10i3.9960

**Published:** 2016-09-13

**Authors:** Sebastián Pita, Francisco Panzera, Pablo Mora, Jesús Vela, Teresa Palomeque, Pedro Lorite

**Affiliations:** 1 Sección Genética Evolutiva, Facultad de Ciencias, Universidad de la República, Montevideo, Uruguay; 2 Departamento de Biología Experimental, Área de Genética, Universidad de Jaén, Jaen, Spain

**Keywords:** Cimicomorpha, kissing bugs, holocentric chromosomes, telomeres, NGS, (TTAGG)_n_

## Abstract

Next-generation sequencing data analysis on *Triatoma infestans* Klug, 1834 (Heteroptera, Cimicomorpha, Reduviidae) revealed the presence of the ancestral insect (TTAGG)_n_ telomeric motif in its genome. Fluorescence *in situ* hybridization confirms that chromosomes bear this telomeric sequence in their chromosomal ends. Furthermore, motif amount estimation was about 0.03% of the total genome, so that the average telomere length in each chromosomal end is almost 18 kb long. We also detected the presence of (TTAGG)_n_ telomeric repeat in mitotic and meiotic chromosomes in other three species of Triatominae: *Triatoma dimidiata* Latreille, 1811, *Dipetalogaster maxima* Uhler, 1894, and *Rhodnius prolixus* Ståhl, 1859. This is the first report of the (TTAGG)_n_ telomeric repeat in the infraorder Cimicomorpha, contradicting the currently accepted hypothesis that evolutionarily recent heteropterans lack this ancestral insect telomeric sequence.

## Introduction

Telomeres, the physical ends of eukaryote chromosomes, are defined as specialized DNA-protein structures essential for chromosome replication, meiotic pairing and chromosome stability. In most organisms, telomeric DNA is composed by simple G-rich sequences repeats that extend for tens of base pairs (bp) as much as 150 kb, depending on the organism. Although telomeric repeats are diverse in their DNA sequence composition among different organisms (Zakian 1995), several taxonomic groups possess highly conserved motifs. Vertebrates, including bony fishes, reptiles, amphibians, and mammals exhibit the (TTAGGG)_n_ repeat (Meyne et al. 1989) while the (TTTAGGG)_n_ sequence appears highly conserved in the plant kingdom (Watson and Riha 2010). Extensive studies in arthropods have revealed that the predominant telomeric sequence is a pentanucleotide sequence repeat (TTAGG)_n_, which has been considered as the ancestral telomeric motif in phylum Arthropoda, including insects (Sahara et al. 1999, Frydrychová et al. 2004, Vítková et al. 2005). However, numerous studies contradict this claim. For example several insect groups do not exhibit this telomeric repeat, such as Diptera, Ephemeroptera, Odonata, Dermaptera, Siphonaptera, Mecoptera, Raphidioptera and parasitic Hymenoptera. In addition, Coleoptera, Neuroptera and Hemiptera orders include species with and without the ancestral (TTAGG)_n_ telomeric motif (Frydrychová et al. 2004, Gokhman et al. 2014, Korandová et al. 2014). In these insect groups, the ancestral telomeric motif is replaced by other alternative telomeric sequences such as (TCAGG)_n_ in some coleopteran species (Mravinac et al. 2011), non-long terminal repeat (LTR) retrotransposons in *Drosophila* Fallén, 1823 (Mason et al. 2008), arrays of long satellite repeats in Culicomorpha dipteran (Walter et al. 2001), or by unknown sequences as in damselflies, mayflies and some aphid species (Frydrychová et al. 2004, Vítková et al. 2005). The most illustrative example of the variability of the telomeric sequences was observed in Coleoptera where ancestral (TTAGG)_n_ has been lost at least eight times during the evolution of this insect group (Frydrychová and Marec 2002, Mravinac et al. 2011).

Among Hemiptera, the ancestral motif is present in the suborder Sternorrhyncha (coccids and aphids with some exceptions) (Mohan et al. 2011, Monti et al. 2011, Novotná et al. 2011), in several genera of Auchenorrhyncha (Frydrychová et al. 2004, Maryańska-Nadachowska et al. 2013, Golub et al. 2014, Kuznetsova et al. 2015a) and Coleorrhyncha (Kuznetsova et al. 2015b) suborders. In the suborder Heteroptera, only two species of the basal infraorders Nepomorpha and Gerromorpha show the ancestral telomeric motif (Kuznetsova et al. 2012, Mason et al. 2016). On the contrary, the most derived and specious heteropteran infraorders (Cimicomorpha and Pentatomomorpha) do not show the classic insect motif (for review see Grozeva et al. 2015, Mason et al. 2016). A recent survey of several sequenced genomes of these groups, including the triatomine *Rhodnius prolixus*, confirms the lack of the ancestral telomeric repeat and these groups are regarded as having a defective version of telomerase gene (Mason et al. 2016). Mason et al. (2016) have suggested the occurrence of a single loss event of the telomeric repeat, sometime before the Cimicomorpha and Pentatomomorpha divergence, and after their separation from Nepomorpha.

Kissing bugs (Triatominae, Reduviidae) are included within the infraorder Cimicomorpha (Heteroptera), constituting a group of medical relevance because they act as vectors of Chagas disease, also known as American trypanosomiasis. This subfamily includes 150 species, of which more than 80 have been cytogenetically studied (Panzera et al. 2010), having holocentric chromosomes. The current data, as above mentioned, suggest a high heterogeneity in insect telomere composition. One should also take into consideration that loss of the insect ancestral repeat in Cimicomorpha has been reported (Mason et al. 2016). For all these reasons it is important to explore for the first time in Triatominae the presence of (TTAGG)_n_ motif, using next-generation sequencing (NGS) analysis tools and fluorescence *in situ* hybridization (FISH) in four triatomine species from three different genera. The results presented in this paper are in clear contradiction to the loss of ancestral telomeric repeats hypothesis in evolutionarily advanced Heteroptera.

## Materials and methods

### Material

Four species where analyzed, involving three different genera from the two principal tribes of the subfamily: Triatomini (*Dipetalogaster maxima*, *Triatoma infestans*, and *Triatoma dimidiata*) and Rhodniini (*Rhodnius prolixus*). The last three species are the main vectors of Chagas disease. Origin and cytogenetic traits of each species are detailed in Table 1.

**Table 1.** Geographical origin and male diploid chromosome number in the four species here analyzed. A = autosomes.

Species	Geographical origin	Male diploid chromosome number (2n)
**Tribe Rhodniini**		
***Rhodnius prolixus***	Guatemala, Quezaltenango, Insectary CDC (USA)	22= 20A + XY
**Tribe Triatomini**		
***Dipetalogaster maxima***	Baja California, Mexico	22= 20A + XY
***Triatoma dimidiata***	Jutiapa, Guatemala	23= 20A + X_1_X_2_Y
***Triatoma infestans***	Tacuarembó, Uruguay	22= 20A + XY

### Telomere detection by genome sequencing

A *Triatoma infestans* (non-Andean lineage) specimen collected in Tacuarembó (Uruguay) was used for sequencing. Approximately 3 µg of genomic DNA were employed in a low coverage Illumina® Hiseq™ 2000 paired-end sequencing. Graph-based clustering analysis was carried out using RepeatExplorer (Novák et al. 2013), implemented within the Galaxy environment (http://repeatexplorer.umbr.cas.cz/) (Novák et al. 2010). RepeatExplorer also allow quantifying the abundance of the repeated sequences in the genome in base to the number of reads in each cluster.

### Telomere detection by FISH

Chromosome preparations for FISH analyses were obtained from male gonads. Testes were removed from live adult insects, fixed in an ethanol–glacial acetic acid mixture (3:1) and stored at -20°C. Squashes were made in a 50% acetic acid drop, coverslips were removed after freezing in liquid nitrogen and the slides were air dried and then stored a 4°C.

Telomeric TTAGG probe generation and FISH assays were carried out following Lorite et al. (2002) and Mora et al. (2015). Telomeric probes were generated by PCR using the primers (TTAGG)_6_ and (TAACC)_6_, following a similar procedure as described by IJdo et al. (1991). PCR was performed in 100 µl using 100 pmol of each primer and 2.5 units of Taq polymerase, in the absence of a template. PCRs were carried out using the following cycling profile: 30 cycles at 95°C (60 sec), 50°C (1 min), 72°C (3 min), with a final elongation step of 72°C for 10 min. PCR generated fragments (between 200 bp and 1 kb) were purified and labeled with biotin-16-dUTP (Roche) out using the Nick Translation Kit (Roche), following manufacturer’s instructions. The labelled probe was precipitated and dissolved in 50% formamide.

Previously to hybridization, slides were treated with RNase A, pepsin and formaldehyde and dehydrated in 70%, 90% and 100% ethanol for 5 min each. Hybridization was performed applying 25 µl of DNA labelled solution to each slide, which was heated for 3 min at 80°C to denature the DNA, and immediately chilled on ice for 3 min. The hybridization mix consisted of (final concentrations) 50% formamide, 2xSSC, 50 mM sodium phosphate, 0.1 mg/ml sonicated salmon sperm DNA, 0.1 mg/ml yeast RNA, and 5 ng/ml labeled telomere probe. The slides were transferred to a moist chamber humidified with formamide (50%) and incubated overnight at 37°C. After incubation, the slides were washed in 50% formamide at 37°C, three times, 3 min each; followed by 2xSSC, 0.05% Tween-20, pH 7.5, three times, 5 min each. Fluorescence immunological detection was performed using the avidin-FICT/ anti-avidin-biotin system with four rounds of amplification. Slides were mounted with Vectashield (Vector). DAPI in the antifade solution was used to counterstain chromosomes.

## Results and discussion

**Figure 1.**
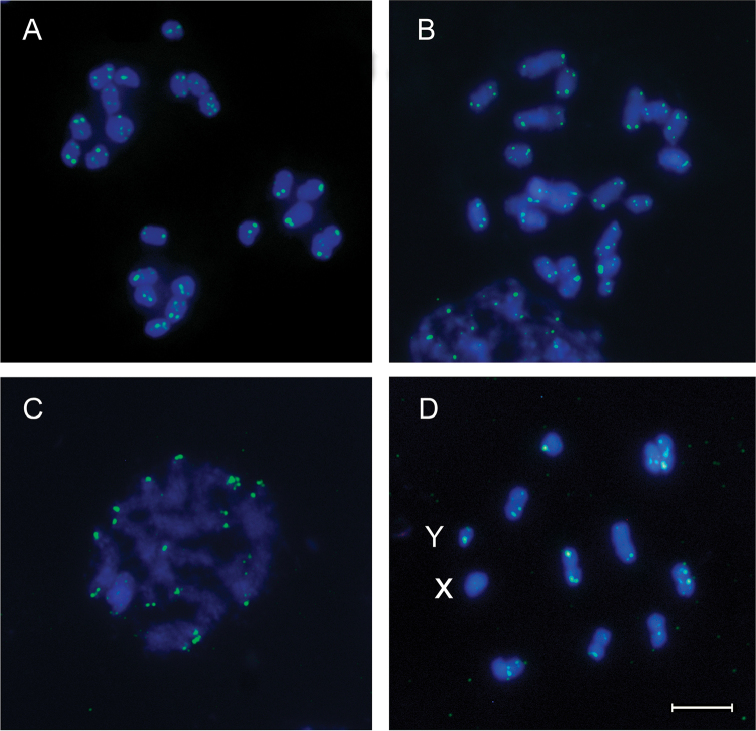
Fluorescence *in situ* hybridization with (TTAGG)_n_ telomeric probe (green signals) on mitotic and meiotic chromosomes (counterstained with DAPI in blue) of four Triatominae species. **A** *Triatoma infestans* (2n=22), spermatogonial prometaphase **B** *Triatoma dimidiata* (2n=23), spermatogonial prometaphase **C** *Dipetalogaster maxima* (2n=22), pachytene stage **D** *Rhodnius prolixus* (2n=22), first meiotic division showing 10 bivalents and two sex chromosomes (X and Y). Scale bar: 5 µm.

The data obtained from the *Triatoma infestans* genome sequencing were analyzed with RepeatExplorer (Novák et al. 2013). One of the obtained clusters was formed by a telomeric sequence TTAGG array. In order to test if this repeat represents the putative telomere, FISH was carried out using the TTAGG repeat as probe. Hybridization signals were clearly seen at the ends of the mitotic chromosomes (Fig. 1A), revealing that telomeres in this species are really composed by this ancestral insect motif. The cluster of the (TTAGG)_n_ sequences was estimated for about 0.0266% of the total genome size, i.e. 395.5 kb. Considering that the haploid genome content in *Triatoma infestans* is 1.52 pg (1.487 Mb) (Panzera et al. 2007, 2010) and that the chromosome number is 2n=22, the average telomeres length motifs in each chromosome end would be almost 18 kb long. This value is in the range of the telomere length observed in other insects with the ancestral motif or a variant of this repeat such as *Tenebrio molitor* Linnaeus, 1758 (15 kb) (Richards et al. 2008) but higher than the observed in other species with holocentric chromosomes as lepidopteran species (6-9 kb) (Okazaki et al. 1993, Mandrioli 2002), or in the homopteran coccid *Planococcus lilacinus* Cockerell, 1905 (6.4 kb) (Mohan et al. 2011).

Furthermore, we tested the telomeric motif presence by FISH in other three triatomine species with (TTAGG)_n_ probe. Hybridization signals were clearly seen on the chromosomal ends of mitotic and meiotic chromosomes (Fig. 1B–D), revealing that Triatominae telomeres are composed by the ancestral insect motif. FISH technique in triatomines is highly sensitive to material fixation conditions. Cytoplasmic remnants in the slides represent the greatest challenge because it hinders the access of the telomeric probes to the chromosomes. This can be partially avoided using recently extracted gonads. In addition, access of the telomeric probes to the chromosome and its visualization are very sensitive to the chromosomes being on the same plane. As a result, differences in hybridization signals can be observed in the same slide or even within chromosomes of the same cell (Fig. 1).

Given our positive FISH hybridization results on *Rhodnius prolixus* chromosomes, we additionally conducted a BLAST search of telomeric sequences in the published genome of this species, available at https://www.vectorbase.org/. Similar as reported by Mason et al. (2016), we did not find (TTAGG)_n_ repeats, so that these tandem sequences and probably others repeated sequences are not included in the published genome of *Rhodnius prolixus* (Mesquita et al. 2015). This reveals the difficulty of the repetitive DNA fraction assembly, as has been reported in different organisms including the well-studied human genome, making that many repetitive sequences have been omitted from the reference assembly and from most genome-wide analyses (Altemose et al. 2014).

Heteroptera or true bugs are a hemipteran suborder comprising seven infraorders and 40,000 species. All phylogenetic studies agreed that the infraorders Cimicomorpha and Pentatomomorpha are the most evolutionarily derived groups, with a common ancestor and involving about 80% of heteropteran species (Weirauch and Schuh 2011). Until now, the detection by FISH, Southern and/or dot-blot hybridization of telomeric repeat motif (TTAGG)_n_ in Heteroptera has been unsuccessful in nine genera from five families of the infraorders Cimicomorpha and Pentatomomorpha (Sahara et al. 1999, Kuznetsova et al. 2011, Frydrychová et al. 2004, Grozeva et al. 2011, Golub et al. 2015). Only two heteropteran species from the basal infraorders Nepomorpha and Gerromorpha exhibit the ancestral telomeric motif (Kuznetsova et al. 2012, Mason et al. 2016). The (TTAGG)_n_ motif was suggested to be lost in the early evolution being and secondarily replaced by another motif or an alternative telomerase-independent mechanism of telomere maintenance (Frydrychová et al. 2004, Lukhtanov and Kuznetsova 2010). Although several authors have suggested the loss of TTAGG repeat in all Cimicomorpha species (Grozeva et al. 2015, Mason et al. 2016), the results presented here clearly contradict this hypothesis. According to the most comprehensive phylogeny of assassin bugs, the subfamily Triatominae is the youngest within Reduviidae, having evolved in the Oligocene, approximately 32 million years ago (24–38 Ma) (Hwang and Weirauch 2012). Whereas, a new acquisition of telomeric repeat in this recent evolutionary group seems unlikely, probably this lack of detection in Cimicomorpha and Pentatomomorpha is due to a methodological problem of the telomeric probe rather than a loss process during their evolution. Detailed analyses of the genomes repetitive fraction as well as exhaustive bioinformatics search on genomic databases might clarify the existence of these repeat sequences in other heteropteran groups.
